# The Role of 8-Oxoguanine DNA Glycosylase-1 in Inflammation

**DOI:** 10.3390/ijms150916975

**Published:** 2014-09-23

**Authors:** Xueqing Ba, Leopoldo Aguilera-Aguirre, Qura Tul Ain Nmi Rashid, Attila Bacsi, Zsolt Radak, Sanjiv Sur, Koa Hosoki, Muralidhar L. Hegde, Istvan Boldogh

**Affiliations:** 1 Department of Microbiology and Immunology, University of Texas Medical Branch, Galveston, TX 77555, USA; E-Mails: baxq755@nenu.edu.cn (X.B.); leaguile@utmb.edu (L.A.-A.); bacsi.attila@gmail.com (A.B.); radak@mail.hupe.hu (Z.R.); 2 Key Laboratory of Molecular Epigenetics, Institute of Genetics and Cytology, Northeast Normal University, Changchun 130024, China; 3 Departments of Internal Medicine, University of Texas Medical Branch, Galveston, TX 77555, USA; E-Mails: qnrashid@utmb.edu (Q.T.A.N.R.); sasur@utmb.edu (S.S.); kohosoki@utmb.edu (K.H.); 4 Institute of Immunology, Medical and Health Science Center, University of Debrecen, Debrecen H-4012, Hungary; 5 Research Institute of Sport Science, Faculty of Physical Education and Sport Science, Semmelweis University, Budapest H-1025, Hungary; 6 Radiation Oncology and Neurology, Houston Methodist Research Institute, Houston, TX 77030, USA; E-Mail: mlhegde@HoustonMethodist.org; 7 Sealy Center for Molecular Medicine, University of Texas Medical Branch, Galveston, TX 77555, USA

**Keywords:** OGG1, DNA base excision repair, 8-oxoG base, small GTPases, inflammation

## Abstract

Many, if not all, environmental pollutants/chemicals and infectious agents increase intracellular levels of reactive oxygen species (ROS) at the site of exposure. ROS not only function as intracellular signaling entities, but also induce damage to cellular molecules including DNA. Among the several dozen ROS-induced DNA base lesions generated in the genome, 8-oxo-7,8-dihydroguanine (8-oxoG) is one of the most abundant because of guanine’s lowest redox potential among DNA bases. In mammalian cells, 8-oxoG is repaired by the 8-oxoguanine DNA glycosylase-1 (OGG1)-initiated DNA base excision repair pathway (OGG1–BER). Accumulation of 8-oxoG in DNA has traditionally been associated with mutagenesis, as well as various human diseases and aging processes, while the free 8-oxoG base in body fluids is one of the best biomarkers of ongoing pathophysiological processes. In this review, we discuss the biological significance of the 8-oxoG base and particularly the role of OGG1–BER in the activation of small GTPases and changes in gene expression, including those that regulate pro-inflammatory chemokines/cytokines and cause inflammation.

## 1. Introduction

The etiology of inflammatory lung diseases is diverse, and often linked to environmental pollutants (generated by natural processes and industrial activities such as heating, oil, chemical processing, vehicle operations, cooking, and tobacco smoking) and exposure to biological agents (pollens, viruses, fungi and/or bacteria). When inhaled, such pollutants primarily impact the surface of the airway (airway lining fluid and epithelia), and when in excess, their interactions with the airway epithelium and resident immune cells usually result in the release of pre-synthesized inflammatory mediators, or gene expression, including that of pro-inflammatory genes, and cause acute inflammation and/or lead to both flare-up and worsening of established diseases (reviewed in [1,2,3]).

Most, if not all, environmental agents elicit the generation of reactive oxygen species (ROS), either directly, indirectly or both, which is among the first cellular responses. ROS operate as intracellular signaling molecules, a function that is commonly documented but whose mechanism is not fully understood [4]. Due to reactivity, ROS modify proteins, lipids, and DNA. In the DNA, one of the most common oxidation products is 8-oxo-7,8-dihydroguanine (8-oxoG) [5]. Oxidatively damaged DNA bases such as 8-oxoG are preferentially repaired by the base excision repair (BER) pathway [6,7]. While oxidative stress causes increased levels of all types of oxidatively modified DNA bases, accumulation of 8-oxoG in the DNA has been specifically linked to various inflammatory disease processes [7,8].

In this review, we discuss the significance/role of genomic 8-oxoG and the 8-oxoguanine DNA glycosylase-1 (OGG1)-generated 8-oxoG base in lung inflammatory processes and inflammation-associated pathologies. We also provide data to support the novel concept of a role for OGG1-generated DNA repair intermediate(s) and repair products in the activation of cellular signaling controlling pro-inflammatory gene expression, and its possible link to acute and chronic inflammation that may be important in lung diseases.

## 2. Results and Discussion

### 2.1. Defense against the Accumulation of 8-oxo-7,8-Dihydroguanine (8-oxoG) in the Genome

The primary ROS generated by biological processes are the superoxide anion (O_2_^−^) and the non-radical oxidant hydrogen peroxide (H_2_O_2_). These reactive species are promptly detoxified, but when in excess, are converted into a hydroxyl radical (·OH), the most reactive oxygen-centered radical [9]. In DNA or RNA, ROS including ·OH primarily damages guanine because it has the lowest ionization potential among nucleic acid bases [10,11,12]. Mechanistic studies show that when, e.g., ·OH interacts with guanine, it results in a reducing neutral radical that reacts with molecular oxygen (O_2_) and, via electron transfer, forms 8-oxoG [13,14,15]. Guanine is preferentially oxidized when it is located at the 5'-end of a series of guanine, often found in promoter/enhancer sequences of various genes and telomeres [16]. Preferential guanine oxidation has been attributed to the migrating radical cation of the guanine, with termination by trapping and forming the final product [10,16]. It is estimated that up to 100,000 8-oxoG lesions could be formed in DNA per cell daily (reviewed in [5]).

To prevent 8-oxoG accumulation and its mutagenic effects, mammalian cells express DNA repair proteins that are analogous to the *Escherichia coli* proteins MutM (formamidopyrimidine DNA glycosylase, also known as Fpg), Nei (endonuclease VIII-like DNA glycosylase), and MutT (8-oxo-7,8-dihydrodeoxyguanosine triphosphate GTPase) [17]. OGG1 is an eukaryotic functional homolog for MutM that excises 8-oxoG and its open-ringed form 2,6-diamino-4-hydroxy-5-formamidopyrimidine (FapyG) from the 8-oxoG/FapyG:C mis-pairing [18]. OGG1 has targeting signals for both mitochondrial import (amino acid residues 9–26) and nuclear localization (amino acids 335–342) [19]. OGG1 is a bifunctional DNA glycosylase with an associated apurinic/apyrimidinic (AP) lyase activity that cleaves DNA at abasic sites via a β-elimination mechanism (reviewed in [17,20]).

**Figure 1:**
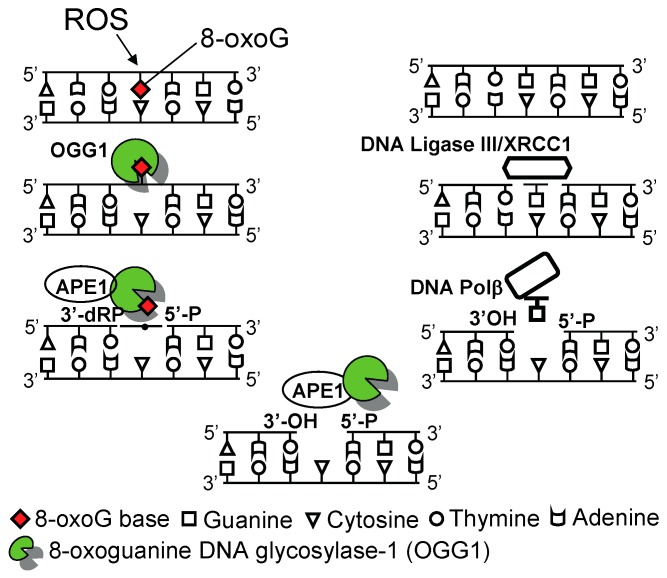
Graphical illustration of 8-oxoguanine DNA glycosylase-1 (OGG1)-initiated genome damage repair. OGG1 is a DNA glycosylase/AP lyase that first hydrolyses the *N*-glycosyl bond releasing 8-oxoG as a free base and subsequently cleaves the sugar-phosphate backbone directly, generating an apurinic/apyrimidinic (AP)-site(s). Apurinic*/*apyrimidinic endonuclease 1 (APE1) processes the single-strand gap by removing the 3'-phospho-α,β-unsaturated aldehyde residue to form a 3'-OH end. DNA polymerase β inserts guanine, and the nicks are ligated by ligase II/XRCC1. AP site, apurinic/apyrimidinic site; APE1, apurinic/apyrimidinic endonuclease1; DNA Polβ, DNA polymerase β; XRCC1, X-ray cross-complementing protein 1.

OGG1-initiated DNA base excision repair pathway (OGG1–BER) is a multistep process, which includes lesion recognition, changes in DNA structure, insertion of the 8-oxoG-containing DNA double helix into the base-binding pocket (active site) of OGG1, and base excision and strand cleavage [17,20,21]. The catalytic site of OGG1 is localized to Lys-249 and Asp-268, and both are in the conserved helix–hairpin–helix (HhH) region of the molecule. HhH is adjacent to a Gly/Pro-rich loop and a conserved aspartic acid motif, which is essential for the glycosylase as well as DNA-binding activity. During repair processes, 8-oxoG is flipped into the OGG1 substrate-binding pocket, and then is inserted between the Phe-319 and Cys-253 and the de-protonated thiolate anion at Cys-253 that stabilizes interactions between the positively charged Lys-249 and nucleobase. The flipped out 8-oxoG base is excised via cleavage of its *N*-glycosidic bond, and 8-oxoG is released from DNA as a free base [21,22].

After 8-oxoG base removal, OGG1, via its AP-lyase activity, cleaves the DNA phosphate backbone at the abasic site by formation of 3'-phospho-α,β-unsaturated aldehyde (3'dRP) and 5'-phosphate termini (called apurinic/apyrimidinic (AP)-site). The 3'-dRP terminus produced by the β-elimination reaction cannot serve as a primer for repair synthesis by DNA polymerases, so it must be removed by a 3'-phosphodiesterase activity of the apurinic*/*apyrimidinic endonuclease 1 (APE1), generating a 1-nucleotide gap [17,20,22]. DNA polymerases, primarily the DNA polymerase β, then incorporate guanine at the gapped site, and the resulting nicks are subsequently sealed by a DNA ligase to complete the repair process (Figure 1).

### 2.2. 8-oxoG Is a Biomarker of Oxidative Stress

#### 2.2.1. 8-oxoG Is a Biomarker of Environmental Lung Exposures

Compelling evidence has established that guanine is the main target for ROS in DNA, with 8-oxoG being the most frequent base lesion [13,14,15]. Therefore, 8-oxoG is considered and routinely used as a biomarker of oxidative damage to DNA [5]. The level of genomic 8-oxoG correlates well with the dose and length of exposure, chemical composition, and physical nature of the inhaled agents/oxidants [23,24,25,26,27,28]. For examples, intrahelical levels of 8-oxoG were significantly increased in lung epithelial resident macrophages, peripheral blood monocytes, and exogenomic 8-oxoG/7,8-dihydro-8-oxo-2'-deoxyguanosine (8-oxodG) levels in body fluids (e.g., serum, urine, sputum, bronchoalveolar lavage (BALF)) upon exposure to environmental pollutants (e.g., various gases: ozone, NO_2_), metals (e.g., lead, cadmium), smoke of various origins, particulate matter (fine, ultrafine particles), chemicals (bisphenol, arsenic, and so on), radiation and others) [26,27,29,30,31,32,33,34,35]. The estimated fold changes in 8-oxoG levels in DNA and its concentration in body fluids were significantly increased, but the extent of these changes varied, primarily due to differences in the sensitivity of the technology used (e.g., high-performance liquid chromatography, reversed-phase liquid chromatography with electrospray tandem mass spectrometry detection and isotope dilution method, Comet assay, or ELISA) [23,24,25,26,27,28,29,30,31,32,33,34,35]. Together, these findings provide strong evidence that environmental lung exposures induce oxidative damage to DNA in the form of increased levels of oxidative bases (and strand lesions), primarily 8-oxoG.

#### 2.2.2. Oxidatively Modified Guanine Lesions Are Signatures of Chronic Lung Inflammation

Inflammation is widely documented in common lung diseases, including asthma, chronic obstructive pulmonary disease (COPD) and other infiltrative pulmonary diseases [36,37,38,39,40,41]. In these disease conditions, oxidative stress is primarily mediated by ROS generated by activated inflammatory cells, which can be enhanced by an increased burden of inhaled environmental agents [42]. ROS, via direct signaling or oxidative modification of lipids, proteins and DNA, are believed to play a vital role in maintaining chronic inflammatory processes [2,3,42]. 8-oxoG is one of the most-documented forms of DNA base damage in the genome of constituent lung cells [43,44,45,46,47,48,49,50] and lungs of animal disease models [51]. Available reports provide strong evidence for the accumulation of 8-oxoG in DNA (and RNA) by inflammatory cell(s)—associated ROS, and ROS induced by environmental lung exposures and 8-oxoG have been acknowledged as an oxidative bio-marker [43,44,45,46,47,48,49,50]. An etiological connection between intrahelical levels of 8-oxoG and inflammatory lung diseases has been proposed; however, it remains unknown whether or not these lesions contribute to of inflammatory processes.

### 2.3. 8-Oxoguanine DNA Glycosylase-1 (OGG1): Role in Inflammatory Processes

#### 2.3.1. Lack of 8-oxoG Repair by OGG1 Confers Resistance to Inflammation in Mouse Model

To study the consequences of 8-oxoG accumulation in DNA and the role of OGG1 in patho-physiological processes, *Ogg1* knockout (*Ogg1*^−/−^) mice were developed in independent laboratories [52,53]. Intriguingly, the lack of OGG1 activity and the consequent supraphysiological levels of genomic 8-oxoG did not affect mouse embryonic development or life span. Under chronic oxidative stress, 8-oxoG levels may increase by 250-fold in *Ogg1*^−/−^ mice without severe consequences, e.g., the liver regenerated to the same extent as it did in untreated *Ogg1*^−/−^ or *Ogg1*^+/+^ mice, and there was no incidence of precancerous lesions or tumors in various organs during a 16-month observation period [54,55]. Similarly, although high levels of 8-oxoG were found in the mitochondrial DNA (>20-fold increase *vs.* wild-type), the mitochondria were functionally normal, and there were no detectable changes in maximal respiration rates or in mitochondrial ROS generation [56]. Unexpectedly, *Ogg1*^−/−^ mice have shown a decreased inflammation upon bacterial infection [57] or challenge with pro-inflammatory agents [58], which may mean that these mice lack component(s) of pro-inflammatory signaling, and there is a role for OGG1 in inflammatory processes.

Szabo and colleagues documented for the first time that when compared to the wild type, *Ogg1*^−/−^ mice are more resistant to lipopolysaccharide (LPS)-induced inflammation and organ dysfunction [58]. The decreased immune response was associated with significantly lower serum chemokine/cytokine levels and prolonged survival after LPS exposure, despite a marked increase in LPS-induced oxidative stress in the lungs, heart, kidneys, and liver. These authors also documented that *Ogg1*^−/−^ mice are markedly protected from type I diabetes induced by streptozotocin. This protection was obvious from decreased hyperglycemia and a low incidence of diabetes, and an increase in β-cell mass. Pancreatic levels of macrophage inflammatory protein 1 α (MIP-1α), tumor necrosis factor alpha (TNF-α) and interleukin (IL)-12 were significantly lower, while basal pancreatic levels of IL-10 were increased in *Ogg1*^−/−^ mice compared to the wild type (*Ogg1*^+/+^) ones [58]. Although these studies did not extend to lung tissue, it is noteworthy that patients with chronic lung inflammation have a greater risk of type 1 and type 2 diabetes and obesity [59,60,61], and raises the possibility that OGG1 itself and/or OGG1–BER could be the link to the innate immune processes associated with these diseases.

#### 2.3.2. Decreased Allergic Immune Responses in the Lungs of *Ogg1* Knockout (*Ogg1*^−/−^) Mice

Li and colleagues [62] documented that, when compared to *Ogg1*^+/+^, ovalbumine (OVA) challenge of *Ogg1*^−/−^ mice causes significantly less inflammatory cell infiltration. A decreased inflammatory response was associated with lower levels of T helper (Th) 1 (TNF-α, IFN-γ, IL-2, and IL-12), and Th2 cytokines (IL-4, IL-6, and IL-10) and IL-17 levels in BALF and lung tissues. This was, in turn, due to decreased signal transducers and activators of transcription-1 (STAT1), STAT3, and STAT6 expression/signaling, and thereby to decreased NF-κB phosphorylation, after OVA challenge. These results are supported by data showing that downregulation of *Ogg1* by siRNA led to lower ROS release and IL-4, IL-2, and IL-17 production, but higher IFN-γ production in lung epithelial cells [62]. The authors concluded that OGG1 may influence airway inflammation via the regulation of oxidative stress (OS).

Substantial amounts of data support the idea that the airway epithelium is the primary cell type in orchestrating an inflammatory response in the lungs [3,63,64]. Therefore, Bacsi and colleagues [65] investigated how OGG1-initiated repair of oxidatively damaged DNA in the airway epithelium impacts innate and allergic immune responses. To do so, RNAi technology was used to ablate OGG1 from the airway epithelium of ragweed pollen grain extract (RWPE)-sensitized animals [66,67,68] (OGG1-deficient: OGG1^D^ mice) before inducing allergic lung inflammation by RWPE challenge. Mice treated with scrambled RNAi were used as controls (OGG1 proficient: OGG1^P^ mice). In these studies, *Ogg1*^−/−^ mice were avoided, as they lack OGG1 activity globally (*i.e.*, in epithelia, dendritic cells, mast cells, macrophages, neutrophils and eosinophils). The results showed that the RWPE challenge-induced allergic inflammatory response was significantly lower in sensitized OGG1^D^ mice, as determined by the expression of Th2 cytokines, number of eosinophils recruited to airways, epithelial metaplasia, and airway hyperresponsiveness (AHR). In contrast, OGG1–BER proficiency in the airway epithelium was coupled to robust innate (from 6–24 h) and late allergic (from 48–144 h) inflammation. The role of OGG1–BER in these processes was specific, as RNAi depletion of the Nei-like glycosylases NEIL1 and NEIL2 that preferentially excise ring-opened purines and 5-hydroxyuracil, respectively, had no significant inhibitory effect. In fact, NEIL2 somewhat increased innate and allergic immune responses. The mechanism of this is being presently investigated.

Studies by Bacsi *et al.* [65] have provided a mechanism as to how OGG1-initiated BER of 8-oxoG is involved in exacerbation of antigen-induced allergic inflammatory processes. OGG1 excises 8-oxoG and FapyG from DNA with high efficiency [20], and, via its lyase activity, cleaves the DNA backbone, transiently generating single-strand gaps [69,70,71]. Under physiological conditions, these gaps are fully repaired in consecutive steps of the BER processes (Figure 1) [20,72]. In response to RWPE challenge, an initial robust innate immune response (from 4 h on, as determined by the recruitment of neutrophils) was observed in OGG1-proficient but not in OGG1-deficient mice. In parallel with neutrophilia, a significant increase was observed in DNA single-strand breaks (DNA SSBs) in the genome of airway epithelial cells. The exact mechanism of DNA SSB formation is unknown; however, the authors speculate that under neutrophil-generated oxidative stress (OS) conditions, OGG1–BER may not be completed, due to an OS-induced imbalance between glycosylase and AP lyase reactions during BER. OS may also compromise OGG1’s binding to apurinic/apyrimidinic (AP) sites, and thereby decrease its effects protecting DNA ends against oxidative erosion and apurinic/apyrimidinic (AP) site-cleaving enzymes, such as endonuclease III-like protein 1 and DNA topoisomerases [69,70,73], leading to SSBs. DNA SSBs induced signaling for the activation of transcription factors (e.g., NF-κB), and consequently, the increased expression of Th2-type inflammatory chemokines/cytokines [74,75,76,77,78,79,80] is well-documented. Bacsi and colleagues concluded that the products of OGG1–BER, including DNA repair intermediate(s) (AP-sites and/or DNA SSBs)-induced signaling, increased the expression of Th2 cytokines and allergic immune responses upon challenge of sensitized OGG1-proficient mice. In contrast, mice without OGG1 in their airway epithelium lacked repair of 8-oxoG, so AP-sites and transient DNA SSBs generated by OGG1 conferred resistance to allergic immune responses. These results support the idea that supra-physiological levels of 8-oxoG in the genomes of airway epithelium may not trigger inflammatory processes in mouse model of lung inflammation.

### 2.4. OGG1-Initiated DNA Base Excision Repair Pathway (OGG1–BER): A Link to Pro-Inflammatory Signaling

#### 2.4.1. OGG1–BER Results in Activation of Small Guanosine Triphosphatases (GTPases)

An implication of OGG1–BER in cell signaling was not evident until recent studies, showing that OGG1 binds free 8-oxoG base with high affinity, and the formed OGG1·8-oxoG complex has guanine nucleotide exchange factor (GEF) activity [81]. The binding constant (*K*_d_) of 0.56 nM indicates tight binding of OGG1 with free 8-oxoG base [81]. These results led us to predict that the product’s binding would inhibit OGG1’s glycosylase activity, as is the case with most enzymes. However, to our surprise, results from additional experiments showed that 8-oxoG stimulated OGG1’s glycosylase activity in a concentration-dependent manner [81], which may mean that 8-oxoG serves as a cofactor for OGG1 by binding to an independent site, and not to the active site pocket in OGG1 as shown previously [21,22]. The binding by OGG1 of 8-oxoG base induced conformational changes (as assessed by OGG1’s intrinsic tryptophan fluorescence) in a concentration-dependent manner [81]. In contrast, 8-oxoG base failed to change tryptophan fluorescence when added to mitochondrial form of OGG1 (OGG1-2a; unpublished data). The free FapyG (or 7,8-dihydro-8-oxodeoxyguanosine; 8-oxodG) substrate of OGG1 in DNA [82] was not bound by OGG1, thereby indicating specificity of OGG1’s binding to an 8-oxoG base [81].

These intriguing observations gained high significance because the 8-oxoG-induced conformational change in the OGG1 molecule allowed its interaction with and activation of small guanosine triphosphatases (GTPases). Specifically, in the presence of 8-oxoG, OGG1 caused replacement of GDP for GTP bound to Kirsten rat sarcoma viral oncogene homolog (K-RAS; RAS: rat sarcoma), neuroblastoma RAS viral oncogene homolog (N-RAS) and Harvey (H)-RAS. Thus, the OGG1·8-oxoG complex functions as a GEF in a manner similar to that of other Ras-activating factors [83,84]. The biological significance of these *in vitro* studies was underlined by those showing that increasing the cellular 8-oxoG level by adding it to cells or activating OGG1–BER rapidly increased RAS–GTP levels in a cell type-specific manner. Activation of RAS led to phosphorylation of the mitogen-activated protein kinase (MAPK) kinase (MEK1/2), and extracellular signal-regulated kinase (ERK1/2), and the latter’s nuclear translocation [81,85].

There is high sequence homology among the RAS and Ras homology (Rho) family of small GTPases (RHO) family of GTPases (Ras-related C3 botulinum toxin substrate 1 GTPase (RAC1), RAC2 and RAC3), and cell division control protein 42 homolog [86], which led us to examine whether the repair of oxidatively damaged DNA by OGG1 and consequent formation of OGG1·8-oxoG complex activate the RHO family member RAC1. Hajas and colleagues reported that the OGG1·8-oxoG complex physically interacts with guanine nucleotide-free and GDP-bound RAC1 protein. This interaction resulted in rapid GDP→GTP, but not GTP→GDP exchange, indicating that OGG1·8-oxoG functions as a prototypic GEF [87]. Luo *et al.* (2014) provided further insights into the biological consequences of OGG1-initiated release of 8-oxoG from DNA [88]. These authors have demonstrated that only OGG1-expressing cells display increased activation of RHO-GTPase in oxidatively stressed cells. These observations were unexpected, as many small GTPases are redox-sensitive, and ROS have an effect similar to that of GEFs in that they modulate guanine nucleotide binding of GTPase, which results in an increase in their GTP-bound form [86]. Taken together, these results imply that OGG1–BER and consequent OGG1·8-oxoG complex formation lead to activation of canonical RAS and RHO family GTPases [81,85,87,88,89]. These results also imply that the 8-oxoG base generated during OGG1–BER may serve as a second messenger, as proposed previously [8].

#### 2.4.2. Activation of Rat Sarcoma (RAS) Family GTPases by OGG1–BER Product 8-oxoG in Lungs

**Figure 2:**
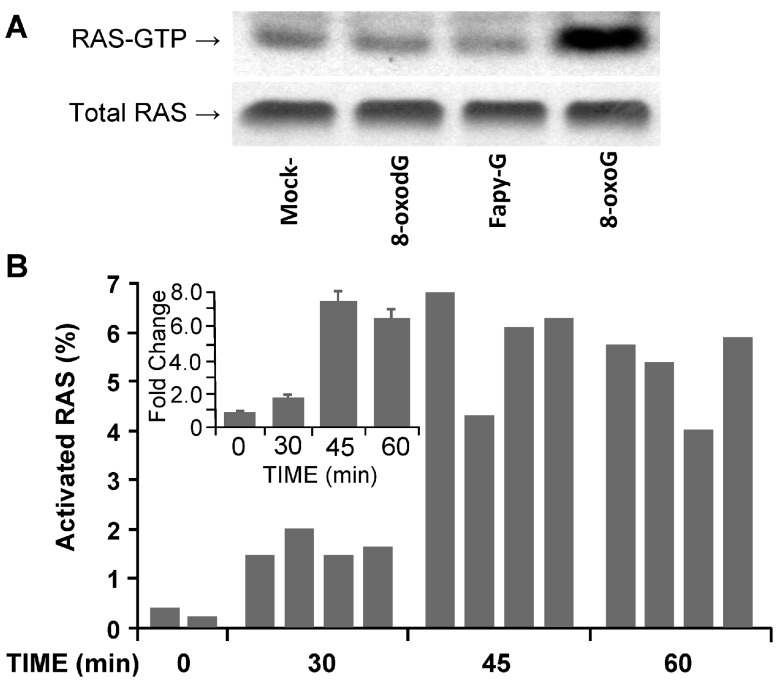
Activation of rat sarcoma–small guanosine triphosphatases (RAS–GTPases) in lungs by 8-oxo-7,8-dihydroguanine (8-oxoG) challenge. (**A**) Challenge of airways with 8-oxoG, but not 2,6-diamino-4-hydroxy-5-formamidopyrimidine (FapyG) or 7,8-dihydro-8-oxo-2'-deoxyguanosine (8-oxodG) (50 ng per kg) increased the GTP-bound levels of RAS (RAS–GTP) levels. Mice (albino, laboratory-bred strain c; Balb/c) were challenged intranasally (i.n.) for 45 min, and lung extracts then prepared for active RAS pull-down assays, as we previously described [81]. Upper panel: RAS–GTP levels in lung extracts of 3–4 individual mice. RAS–GTP was captured by a RAS pull-down assay from 160 μg of lung extract and immunoblotted using Pan-RAS antibody (Ab). Lower panel: Abundance of total RAS in 20 μg per lane of lung extracts, as shown by pan-RAS Ab. 8-oxoG, 8-oxo-7,8-dihydroguanine; FapyG, 2,6-diamino-4-hydroxy-5-formamido-pyrimi-dine; 8-oxodG, 7,8-dihydro-8-oxo-2'-deoxy-guanosine; (**B**) Time course of RAS–GTPase activation after 8-oxoG challenge of lungs. Individual mice were challenged with 8-oxoG (50 ng mg per kg, dose is determined in preliminary studies) i.n. and GTP-bound RAS was determined by ELISA pull-down assays [105]. RAS–GTP was captured from 160 μg of lung extract. The percentages of increase were calculated using MS Excel. The abundance of total RAS was determined in 20 μg of lung extract by ELISA pull-down assays. Animal experiments were performed according to the NIH Guide for the Care and Use of Experimental Animals and approved by the UTMB IACUC (no. A0807044).

To test for the link between OGG1·8-oxoG and RAS activation, as well as increased pro-inflammatory chemokine/cytokine expression and inflammation, mouse lungs were challenged intranasally with 8-oxoG base (50 ng per kg, as determined in preliminary studies). At various times thereafter, lung extracts were prepared and activated levels of RAS–GTPase examined by an active RAS pull-down assay. Challenging the lungs with 8-oxoG base resulted in activation of RAS–GTPase as shown in Figure 2A (the 45-min time point is shown). OGG1 also excises FapyG [6,90], so we challenged lungs with FapyG (50 ng per kg). As shown in Figure 2A, FapyG failed to increase the GTP-bound levels of RAS (RAS-GTP). Similarly, the free 8-oxodG (50 ng per kg) failed to activate RAS GTPase, in line with its inability to bind OGG1 [81] or activate RAC1 and RHO member A GTPases [87,91,92]. Figure 2B shows a time-dependent increase in the percentage of activated RAS–GTP levels in the lungs of individual mice as determined by enzyme-linked immunosorbent assay (ELISA). A summary of RAS–GTP levels in experimental groups is shown in the inset to Figure 2B. Although RAS activation after exposure of cultured cells to 8-oxoG has been documented [81], this is the first detailed analysis of RAS activation in the lungs. These results also imply that the 8-oxoG, when installed into airways, penetrated the airway lining fluid and was taken up by cells of the airway epithelium in a manner similar to that of cultured cells [81,93].

Utilizing cultured cells, previous studies showed that activation of RAS by OGG1·8-oxoG induces downstream signaling, e.g., MEK1/2, and ERK1/2 [81,85]. To further these observations, the findings from microarray analysis of gene expression (accession number: NCBI, GEO #GSE26813) were analyzed further by Ingenuity Pathway Analysis software. The results showed that eight of the top 10 pathways that responded to 8-oxoG exposure involved the small G protein RAS [81]. These observations are in agreement with those showing that RAS–GTPase-induced downstream signaling involves MAPKs and phosphoinositide 3-kinase (PI3K) [94,95], and that signals from the RAS GTPases affected many major cellular processes, including those driving inflammation in pulmonary diseases [95,96,97,98,99].

The relevance of RAS–GTPase and downstream signaling in lung inflammation is underlined by studies using pharmacological inhibitors of RAS and RAS-induced pathways. For example, the *Ras* antagonist, farnesylthiosalicylic acid *(*FTS), was found to be beneficial in lowering autoimmune, acute and chronic inflammatory processes [100]. Similarly, the MAPKK kinase (Raf-1) inhibitor GW5074 and the MEK1,2 inhibitor U0126 both significantly decreased allergic immune responses in a mouse asthma model [101]. The Raf-1 inhibitor GW5074 was effective in preventing smoke-induced AHR in mice [102]. PD098059, a MEK1,2 inhibitor, inhibited LPS-induced release of TNF-α and IL-6 from both monocytes and lung macrophages [103]. The PI3K inhibitor LY294002 decreased airway eosinophilia, mucus hypersecretion, cytokine levels and AHR [101], and also prevented chemokine-induced, sustained cell adhesion *in vivo* [104].

#### 2.4.3. Pro-Inflammatory Gene Expression in the Lungs upon Challenge with 8-oxoG Base

Increased 8-oxoG levels in the serum, urine, and sputum of individuals exposed to environmental pollutants or of asthmatic and COPD patients are considered biomarkers of ongoing inflammatory processes (see above). *In vivo*, the 8-oxoG base can be generated specifically by OGG1-initiated BER [20]. Although proven by fluorescence spectroscopy *in vitro* [81], we propose that when added to the cells 8-oxoG is internalized and can form a functional complex with OGG1 *in cellulo* before being excreted from the cells into the extracellular milieu. This hypothesis is supported by data showing that in OGG1 activity-deficient mice, which do not generate the 8-oxoG base, innate and allergic inflammation are decreased after challenge with LPS, OVA, or RWPE [58,62,65].

**Figure 3:**
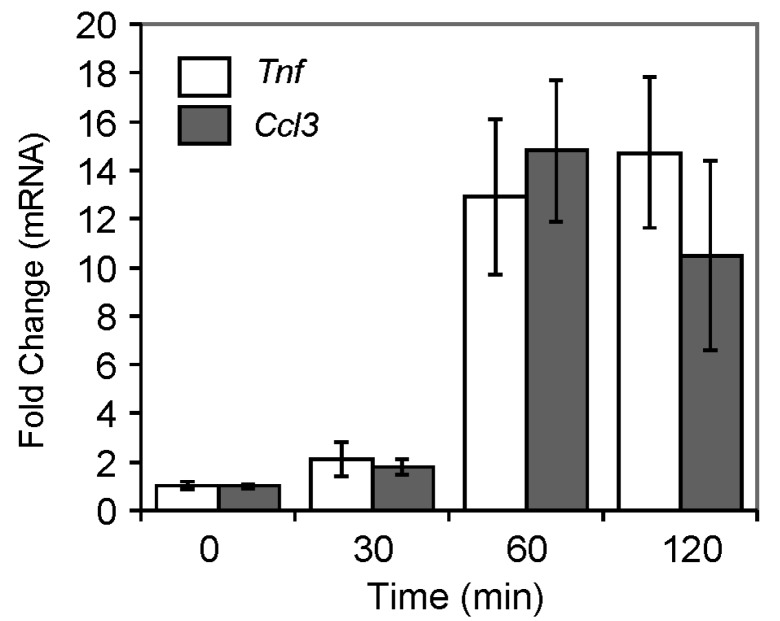
Expression of inflammatory mediators induced by 8-oxoG base challenge. Eight-week-old female Balb/c mice were used for these studies. Mice (*n* = 5–6) were challenged i.n. under mild anesthesia with 60 µL of pH (7.4) balanced saline solution containing 50 ng per kg 8-oxoG, or only saline [68]. After 0, 30, 60 and 120 min of exposure, lungs were removed, and RNAs were isolated. After reverse transcription, mRNA levels of selected cytokines were determined using individual primer pairs. qRT-PCR was performed in an ABI7000 Sequencer, and expression levels were calculated by the ΔΔ*C*_t_ method [106,107]. Animal experiments were performed as described in the legend to Figure 2.

To test the role of OGG1–BER in an immune response, mouse lungs were challenged with the repair product, 8-oxoG base, as described in the legend to Figure 2, and changes in mRNA levels were determined. In preliminary experiments, mRNA levels of tumor necrosis factor (*Tnf*) and chemokine(C–C motif) ligand 3 (*Ccl3*) were examined using appropriate primers with qRT-PCR. As shown in Figure 3, *Ccl3* (encoding the chemokine CCL3, also called macrophage inflammatory protein 1 alpha), and *Tnf* mRNA levels were increased from 30 min on, reaching their maximum after 60–120 min of challenge. We also examined their expression at the protein level after 120 min of 8-oxoG exposure. We found that protein levels of TNF-α and CCL3 were increased 10,600 and 9200-fold, respectively, compared to the mock-treated control. These mediators individually or in combination are potent inducers of pro-inflammatory gene expression [36]. Therefore, a more comprehensive analysis of gene expression at mRNA levels of pro-inflammatory mediators induced by 8-oxoG challenge was restricted to 120 min.

**Figure 4:**
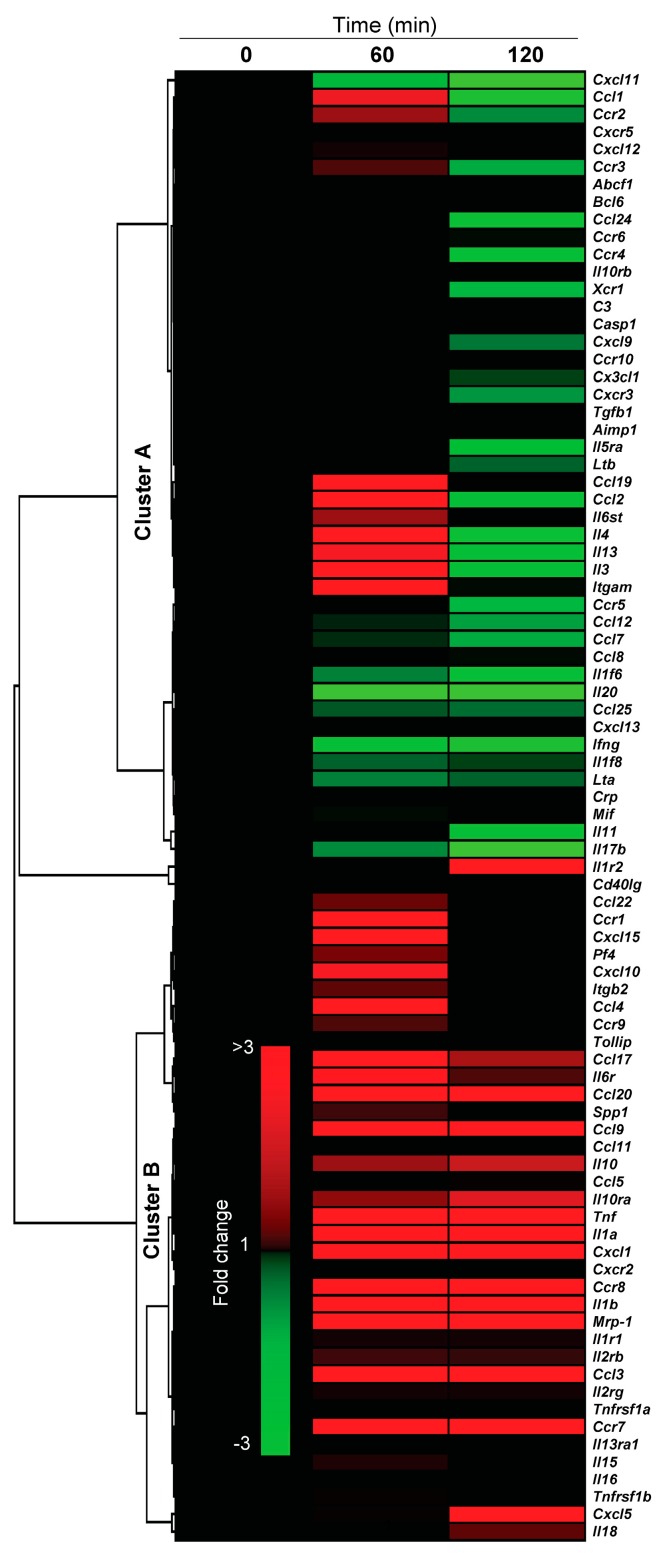
Changes in pro-inflammatory gene expression induced by 8-oxoG, the product of OGG1-BER. Mice were challenged i.n. with 8-oxoG base (50 ng per kg), and RNA was extracted. Total RNA (pooled from five mice) was reverse-transcribed into cDNA and analyzed using a Mouse Inflammatory Cytokines & Receptors PCR Array (Cat # PAMM-011A, SABiosciences, Valencia, CA, USA). Data were analyzed using RT^2^ profiler PCR data analysis template version 2.0. Heat maps were created and unsupervised hierarchical clusters were constructed using GENE-E online software (Broad Institute; Cambridge, MA, USA). Genes were clustered using Spearman’s rank correlation coefficient [108]. The colors in the heat-map show the minimum and maximum values (±3). Animal experiments were performed as described in the legend to Figure 2.

**Figure 5:**
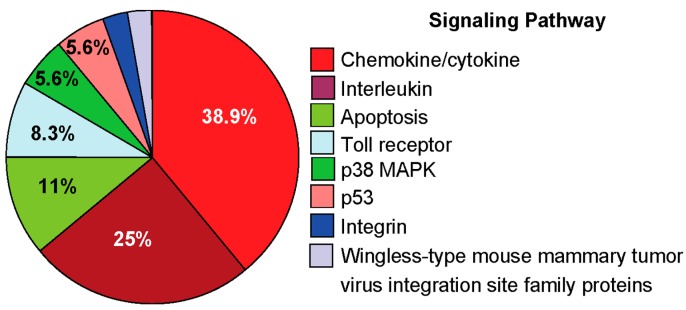
Signaling pathways induced by exposing the lungs to 8-oxoG base. Analysis by PANTHER software included genes that were upregulated in Cluster A and B (Figure 4). The percentages of genes for each category were calculated as described previously [108].

Next, mice were challenged with 8-oxoG base (50 ng per kg), lungs were harvested, and RNAs were isolated after 0, 60 min and 120 of challenge. Purified RNAs from five mice per time point were pooled and reverse transcribed, and the cDNAs subjected to analysis. RNA levels of pro-inflammatory mediators were determined by using an RT² Profiler PCR Array containing 84 key genes (Mouse Inflammatory Cytokines & Receptors PCR, SABiosciences, Valencia, CA, USA). The results are summarized in Figure 4, Figure 5 and Table 1. Figure 4 shows changes in RNA levels of C–C motif and C–X–C motif chemokines, their receptors, interleukins and cytokines. Unsupervised hierarchical clustering divided the 84 genes into two major clusters. Cluster A contained genes having increased expression of *Ccl1*, *Ccl19*, *Ccl2*, *Il6st*, *Il4*, *Il13*, *Il3* and *Igam* only at 60 min (1.8–2.1-fold) and unaltered, or downregulated (e.g., *Cxcl111*, *Ccl12*, *Ccl24*, *Il20*, *Ifng*, *Il17b*) at all time points. Cluster B contains 37 genes out of which 19 genes were upregulated (>2-fold, also shown in Table 1). Genes with increased expression include C–C motif (*Ccl17*, *Ccl19*, *Ccl20*, *Ccl3*, *Ccl4*, *Ccl6*, *Ccl9*), and C–X–C motif chemokines (*Cxcl1*, *Cxcl5*) and *Tnf*. In addition to chemokines/cytokines, 8-oxoG challenge of lungs increased the expression of chemokine receptors, including *Ccr1*, *Ccr7* and *Ccr8*. As shown in Table 1, *Cxcl1* expression is the highest (>65-fold) among the genes included in the array, followed by *Ccl3* (>10-fold), *Ccl20* (>7-fold), *Tnf* (<9-fold), *IL1a* (>5-fold) and *IL1b* (>4-fold). We note that mRNA levels for these chemokines and cytokines could actually be higher at the site of exposure in the airway epithelia, as RNAs were isolated from the whole lungs.

For further evaluation, genes altered significantly at 60 min post-exposure were subjected to PANTHER analysis to identify and rank the signaling pathways [108]. The results from this analysis showed that the OGG1–BER product 8-oxoG base induced several pathways, including inflammation mediated by chemokines and cytokines (38.9%; Panther (P) # p00031), interleukin (25%, P #p00036), and apoptosis signaling (11%, P # p00006). Analysis also showed modulation of Toll receptor (8.3% P # p00054), p38 MAPK (5.6% P # p00018), p53 (5.6% P # p00059), and integrin as well as wingless-type mouse mammary tumor virus integration site family proteins signaling (2.9%).

**Table 1:** Expression of C–C, C–X–C, interleukins (ILs) and TNF in 8-oxoG-challenged lungs.

Symbol	RefSeq ID	Name	60 min	120 min
			Fold Change	
***Ccl17***	NM_011332	Chemokine (C–C motif) ligand 17	2.25	1.62
***Ccl19***	NM_011888	Chemokine (C–C motif) ligand 19	2.28	−1.04
***Ccl20***	NM_016960	Chemokine (C–C motif) ligand 20	7.30	5.02
***Ccl3***	NM_011337	Chemokine (C–C motif) ligand 3	10.53	10.53
***Ccl4***	NM_013652	Chemokine (C–C motif) ligand 4	2.16	1.02
***Ccl6***	NM_009139	Chemokine (C–C motif) ligand 6	2.00	1.97
***Ccl9***	NM_011338	Chemokine (C–C motif) ligand 9	2.09	2.22
***Ccr1***	NM_009912	Chemokine (C–C motif) receptor 1	2.03	1.29
***Ccr7***	NM_007719	Chemokine (C–C motif) receptor 7	2.50	2.28
***Ccr8***	NM_007720	Chemokine (C–C motif) receptor 8	2.76	2.84
***Cxcl1***	NM_008176	Chemokine (C–X–C motif) ligand 1	41.27	65.21
***Cxcl5***	NM_009141	Chemokine (C–X–C motif) ligand 5	1.32	2.47
***IL1*α**	NM_010554	Interleukin 1 α	4.09	5.29
***IL1β***	NM_008361	Interleukin 1 β	4.31	4.40
***IL1r2***	NM_010555	Interleukin 1 receptor, type II	−1.07	2.01
***IL3***	NM_010556	Interleukin 3	2.28	−2.12
***IL4***	NM_021283	Interleukin 4	2.10	−2.47
***Itgam***	NM_008401	Integrin alpha M	2.01	−1.26
***Tnf***	NM_013693	Tumor necrosis factor	7.90	9.93

Importantly, chemokine/cytokine and interleukin signaling pathways account for more than 60% (63.9%) of the total genes activated by OGG1–BER product 8-oxoG, which may mean that 8-oxoG challenge induced primarily an inflammatory response.

**Figure 6:**
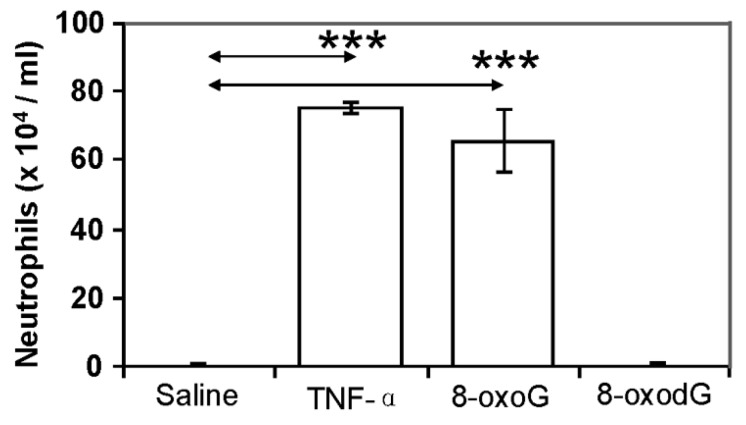
The OGG1–BER product 8-oxoG induces neutrophil recruitment to the lungs. Mice (*n* = 5–6) were challenged i.n. with 60 µL of pH-balanced 8-oxoG solution (pH: 7.4; 50 ng per kg). TNF-α (20 ng per lung) and 8-oxodG (50 ng per kg) and solvent (saline) were used as controls. Bronchoalveolar lavage fluid was collected after 16 h of challenge, cells on cytospin slides were stained with Wright-Giemsa, and the number of neutrophils was counted, as we previously described [68]. All animal experiments were performed as described in Figure 2. *** *p* < 0.001. TNF-α, tumor necrosis factor–α; 8-oxoG, 8-oxo-7,8-dihydroguanine; 8-oxodG, 7,8-dihydro-8-oxo-2'-deoxyguanosine.

To test whether increased expression of chemokines/cytokines/interleukins translated into actual lung inflammation, mice were challenged i.n. with 8-oxoG base (50 ng per kg) [68]. In controls, mice were challenged with TNF-α (20 ng per lung, positive control), 8-oxodG (50 ng per kg), or saline. After 16 h of challenge, lungs were lavaged, and the numbers of inflammatory cells in BALFs were assessed. As shown in Figure 6, 8-oxoG base challenge induced the recruitment of neutrophils into airways. Although the doses of TNF-α and 8-oxoG used for challenge may not be comparable, there were no significant differences between the number of neutrophils recruited by TNF-α [109] and 8-oxoG base challenge. In the lungs of 8-oxodG-challenged mice, there were only background levels of neutrophils.

**Figure 7:**
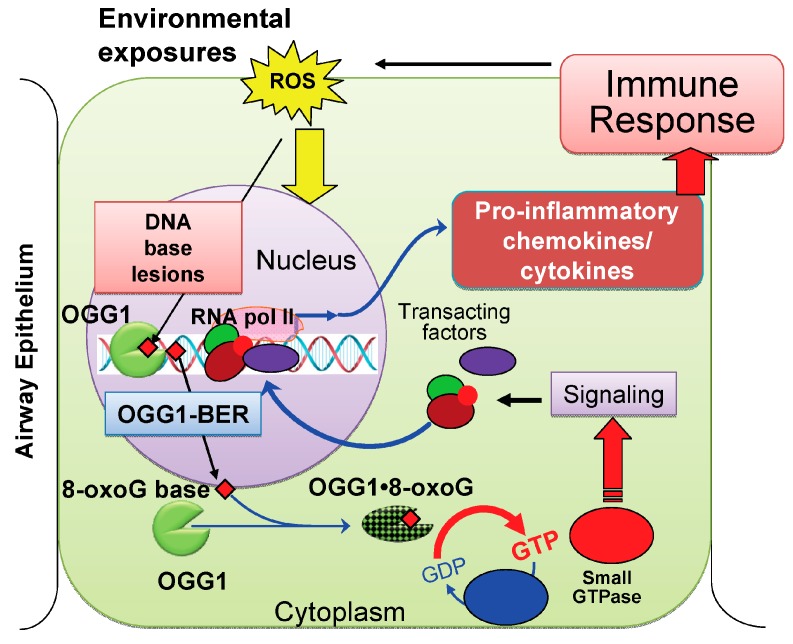
A proposed model for lung inflammation by repair of oxidatively damaged DNA via OGG1–BER. When environmental exposures occur or during an inflammatory process, Reactive oxygen species (ROS) are generated by cellular oxido-reductases and mitochondrial dysfunction in the airway epithelium. Due to guanine’s lowest oxidation potential, the most frequent base damage in DNA is 8-oxoG, which needs to be repaired to prevent mutations. 8-OxoG base is released by OGG1–BER (Figure 1) and transported to the cytoplasm, where it is bound by OGG1, and the resulting complex (OGG1·8-oxoG) functions as a guanine nucleotide exchange factor and mediates GDP→GTP exchange. Activated small GTPase(s) signal expression of pro-inflammatory genes, which induce an innate immune response. Recruited inflammatory cells induced ROS, DNA damage and repair that may lead to a vicious cycle of OGG1·8-oxoG→RAS–GTP→inflammatory signaling, maintaining chronic inflammation.

These results suggested to us that the 8-oxoG base was taken up by the epithelium. When internalized, 8-oxoG possibly formed a complex with OGG1 as proposed previously [81]. The OGG1·8-oxoG complex then serves as a guanine nucleotide exchange factor to mediate GDP→GTP exchange. Activated RAS GTPases induce downstream signaling pathways leading to chemokine/cytokine/IL expression, and consequently an innate inflammatory response. ROS-induced guanine oxidation to 8-oxoG and the repair of this mutagenic base lesion by OGG1–BER are among the first events upon exposure to environmental agent(s). Thus, it is reasonable to propose that OGG1–BER could be an interface between the environment and cell activation signaling for pro-inflammatory gene expression (Figure 7). Although further studies are required, it is proposed that OGG1·8-oxoG→RAS signaling resulting in the expression of inflammatory mediators such as CCL17, CCL19, CCL20, CCL3, CCL4, CCL6 or CCL9 and interleukins could modulate eosinophils, basophils, mast cells, and T and B cell function(s) and thus may also affect the adaptive immune system (Th2, regulatory T, or Th17 cells). Intriguing previous studies [91,92] showed that 8-oxodG suppressed allergy-associated immune responses and tissue remodeling in a mouse model. Another study documented that 8-oxoGTP antagonized the superoxide anion generation of human neutrophils. The exact mechanism by which 8-oxodG or 8-oxoGTP suppresses inflammatory response and inhibit structural changes in lungs as well as neutrophil’s respiratory burst is not known. It is possible that both 8-oxodG and 8-oxoGTP are bound by OGG1 and form such a complex that inhibits GDP→GTP exchange and thus may counteract the pro-inflammatory effects of 8-oxoG. 

## 3. Conclusions

Environmental exposures and an increase in the genomic 8-oxoG level have been associated with inflammatory lung diseases. Intriguing recent studies have shown that a decrease in OGG1 activity markedly prevented chemokine/cytokine expression and a cellular inflammatory response. Based on previous studies and the data shown in this paper, it is evident that OGG1-initiated DNA BER led to the generation of an OGG1•8-oxoG complex that functions as a guanine nucleotide exchange factor “fueling” the activation of small GTPases, which generate cell activation signals for increased expression of pro-inflammatory chemokines/cytokines leading a full-blown inflammatory response. OGG1–BER-mediated inflammation could be an important physiological (beneficial) response aimed at host protection upon environmental challenge. However, it is also possible that when the challenge of the host occurs in association with pre-existing conditions (e.g., immune-suppression, allergy, age or genetic background) where inflammation is not properly controlled, OGG1–BER-driven innate signaling may exacerbate ongoing diseases and aging processes. In addition, the data provided in this review point to a novel mechanism—the potential role of OGG1–BER in the maintenance of chronic inflammation, not only in the lungs, but also in other systemic diseases—such as cardiovascular, arthritis, cancer, dementia, obesity, and diabetes.

## Abbreviations

8-oxoG: 8-oxo-7,8-dihydroguanine
8-oxodG: 7,8-dihydro-8-oxo-2'-deoxyguanosine
AE: airway epithelia
AHR: airway hyperresponsiveness
APE1: apurinic*/*apyrimidinic endonuclease 1
AP sites: apurinic/apyrimidinic site; BER, base excision repair
BALF: bronchoalveolar lavage fluid
CXCL: C–X–C-motif containing chemokine ligands *Cxcls,* gene or mRNA encoding CXCLs
COPD: chronic obstructive pulmonary disease
DC: dendritic cells
FapyG: 2,6-diamino-4-hydroxy-5-formamidopyrimidine
GEF: Guanine nucleotide exchange factor
ERK1/2: extracellular signal-regulated kinase 1/2
H-Ras and K-Ras: mammalian homolog of Harvey and Kirsten sarcoma virus oncogene
IIR: innate immune response
IL: interleukin
LPS: lipopolysaccharides
N-Ras: Neuroblastoma RAS viral oncogene homolog
MAPK: mitogen activated kinase(s)
MEK1/2: mitogen-activated kinase, kinase 1/2
OGG1: 8-oxoguanine DNA glycosylase-1
OGG1^D^: deficient in OGG1 expression
OGG1^P^: Proficient in OGG1 expression
OS: oxidative stress
RHO: Ras homology (Rho) family of small GTPases
RAC1: Ras-related C3 botulinum toxin substrate 1 GTPase
ROS: reactive oxygen species
PI3K: phosphatidyl inositol 3 kinase
RWPE: ragweed pollen grain extract
SSBs: DNA single-strand breaks
